# Physical association of low density lipoprotein particles and extracellular vesicles unveiled by single particle analysis

**DOI:** 10.1002/jev2.12376

**Published:** 2023-11-09

**Authors:** Estefanía Lozano‐Andrés, Agustin Enciso‐Martinez, Abril Gijsbers, Andrea Ridolfi, Guillaume Van Niel, Sten F. W. M. Libregts, Cláudio Pinheiro, Martijn J. C. van Herwijnen, An Hendrix, Marco Brucale, Francesco Valle, Peter J. Peters, Cees Otto, Ger J. A. Arkesteijn, Marca H. M. Wauben

**Affiliations:** ^1^ Department of Biomolecular Health Sciences, Faculty of Veterinary Medicine Utrecht University Utrecht The Netherlands; ^2^ Department of Cell and Chemical Biology Leiden University Medical Center Leiden The Netherlands; ^3^ Medical Cell Biophysics Group University of Twente Enschede The Netherlands; ^4^ Maastricht Multimodal Molecular Imaging Institute, Division of Nanoscopy Maastricht University Maastricht The Netherlands; ^5^ Department of Physics and Astronomy and LaserLaB Amsterdam Vrije Universiteit Amsterdam Amsterdam The Netherlands; ^6^ Institute for Psychiatry and Neuroscience of Paris Hopital Saint‐Anne, Université Descartes Paris France; ^7^ Laboratory of Experimental Cancer Research Department of Human Structure and Repair Ghent University Ghent Belgium; ^8^ Cancer Research Institute Ghent Ghent Belgium; ^9^ Institute for the Study of Nanostructured Materials (ISMN) Italian National Research Council (CNR) Bologna Italy

**Keywords:** atomic force microscopy, biomarker, blood, cryo‐electron tomography, exosomes, extracellular vesicles, flow cytometry, lipoprotein particles, microvesicles, plasma, Rayleigh and Raman scattering, single particle

## Abstract

Extracellular vesicles (EVs) in blood plasma are recognized as potential biomarkers for disease. Although blood plasma is easily obtainable, analysis of EVs at the single particle level is still challenging due to the biological complexity of this body fluid. Besides EVs, plasma contains different types of lipoproteins particles (LPPs), that outnumber EVs by orders of magnitude and which partially overlap in biophysical properties such as size, density and molecular makeup. Consequently, during EV isolation LPPs are often co‐isolated. Furthermore, physical EV‐LPP complexes have been observed in purified EV preparations. Since co‐isolation or association of LPPs can impact EV‐based analysis and biomarker profiling, we investigated the presence and formation of EV‐LPP complexes in biological samples by using label‐free atomic force microscopy, cryo‐electron tomography and synchronous Rayleigh and Raman scattering analysis of optically trapped particles and fluorescence‐based high sensitivity single particle flow cytometry. Furthermore, we evaluated the impact on flow cytometric analysis in the presence of LPPs using in vitro spike‐in experiments of purified tumour cell line‐derived EVs in different classes of purified human LPPs. Based on orthogonal single‐particle analysis techniques we demonstrate that EV‐LPP complexes can form under physiological conditions. Furthermore, we show that in fluorescence‐based flow cytometric EV analysis staining of LPPs, as well as EV‐LPP associations, can influence quantitative and qualitative EV analysis. Lastly, we demonstrate that the colloidal matrix of the biofluid in which EVs reside impacts their buoyant density, size and/or refractive index (RI), which may have consequences for down‐stream EV analysis and EV biomarker profiling.

## 1 INTRODUCTION

Extracellular vesicles (EVs) are a heterogeneous group of membrane enclosed vesicles that contain biological information from the cell of origin, such as lipids, nucleic acids, carbohydrates and proteins, and are involved in intercellular communication. EVs present in blood plasma can be obtained via minimally invasive methods and have been proposed to hold clinical potential as biomarkers for diagnosis and prognosis of diseases since their specific makeup reflects a unique signature of the cell of origin (Boukouris & Mathivanan, 2015; Fais et al., 2016; Théry et al., 2009). Next to small EVs (50–200 nm), plasma contains large amounts of lipoprotein particles (LPPs). LPPs are heterogeneous particles enclosed by a single layer of phospholipids and are often categorized based on their density and protein/lipid composition. The major LPP‐types are chylomicrons (CM), very low‐density lipoprotein (VLDL) particles, low‐density lipoprotein (LDL) particles and high‐density lipoprotein (HDL) particles (Feingold & Grunfeld, 2000). CM have a size range of 75–1200 nm in diameter, with varying concentration between individuals and (fatty) meal consumption. VLDL are derived from CM, with 30–80 nm in diameter, which can be further transformed into LDL particles, with a smaller size diameter range of 5–35 nm. These LPPs have a low density (<0.930–1.063 g/cm^3^) and are reported to contain copies of ApoB proteins (Alonzi et al., 2008; Feingold & Grunfeld, 2000). HDL particles are even smaller in diameter (size range 5–12 nm), have a high density (1.063–1.210 g/cm3) and do not contain ApoB proteins but ApoAI proteins (Alonzi et al., 2008; Feingold & Grunfeld, 2000). Recent studies have shown that the presence of LPPs is expected to interfere with both EV isolation and analysis, as they not only outnumber EVs by several orders of magnitude but also partially overlap in biophysical properties such as size, density and molecular makeup (Botha et al., 2022; Karimi et al., 2018; Simonsen, 2017; Sódar et al., 2016; Yuana et al., 2014). The most widely applied EV isolation methods, that is, differential (ultra)centrifugation, size‐exclusion chromatography (SEC) and density gradient centrifugation, are unable to efficiently separate EVs from LPPs and co‐isolation or formation of co‐precipitates in the final preparations have been reported (Baranyai et al., 2015; Linares et al., 2015; Simonsen, 2017; Sódar et al., 2016; Takov et al., 2018; Yuana et al., 2014). SEC is currently applied frequently for the analysis of clinical samples and although SEC allows separation of EVs and smaller HDL particles, EV‐enriched SEC fractions still contain similarly sized ApoB+ particles (Karimi et al., 2018). This is in agreement with recent studies showing co‐isolation between EVs and LPPs in fresh and processed blood plasma samples (Sódar et al., 2016; Yuana et al., 2013, 2014). The combination of different EV isolation methods can strongly reduce the amount of LPPs and can be applied to obtain EVs from blood plasma with high purity, however such procedures may also result in the selection of certain subpopulations of EVs (Karimi et al., 2018; Onódi et al., 2018; Vergauwen et al., 2017, 2021; Tulkens et al., 2020). Moreover, implementation of such combined EV isolation methods is currently limited in a clinical setting, because these procedures are elaborate, time‐consuming and expensive. Importantly, the fact that EV‐LPP complexes have been detected in EV preparations should be taken into account, since EV characteristics will be influenced in such complexes (Sódar et al., 2016; Yuana et al., 2014). Currently, it is not known whether EV‐LPP complexes are artefacts resulting from the EV isolation methods used, or also occur in a physiological situation. Interestingly, some studies using minimal sample processing reported the presence of LPPs in close proximity to the lipid bilayer of EVs, indicating that EV‐LPP interactions might indeed happen under physiological conditions (Sódar et al., 2016; Yuana et al., 2013, 2014). Overall, this complex biological landscape complicates EV isolation and single EV‐based analysis and hampers the translation of EVs as biomarkers for diseases. Therefore, a better understanding on how the presence of distinct types of LPPs can affect the appearance of EVs is critical for the characterization of single EVs and their use as biomarkers in blood plasma. Techniques that allow for single particle analysis of heterogeneous EV populations are pivotal for this. For example, atomic force microscopy (AFM) morphometry can be employed to simultaneously determine diameter and stiffness of individual particles (Ridolfi et al., 2020), thus making it possible to distinguish between EVs and LPPs in complex mixed samples on the basis of their nanomechanical properties (Ridolfi et al., 2022). Cryo‐electron tomography (ET), although not being high‐throughput, allows for the visualization of heterogeneous samples with great resolution and is able to distinguish between lipid bilayer EVs and single layer LPPs (Cizmar & Yuana, 2017). Label‐free synchronous Rayleigh and Raman scattering analysis of optically trapped particles has been proposed as a feasible approach to detect and differentiate both EVs and LPPs based on their Raman spectrum and molecular composition with minimal need for sample processing (Enciso‐Martinez, Van Der Pol, Hau et al., 2020). Flow cytometry (FC) is a high‐throughput multiparametric technique that is widely incorporated into clinical labs. However, detection of single EVs is challenging due to the resolution limit of most available instruments and the intrinsic features of EV, such as their small size and low refractive index (RI) (Nolan, 2015; van der Vlist et al., 2012). Previous studies have shown that the presence of LPPs in plasma can influence light scatter‐triggered FC detection of EV (Sódar et al., 2016; van der Pol et al., 2018). Fluorescence‐triggered FC is an alternative for detection of EVs but depends on fluorescent staining procedures, for example, staining with generic fluorescent dyes and/or incubation with fluorophore‐conjugated antibodies against specific proteins (Arraud et al., 2016; Morales‐Kastresana et al., 2017; Nolan, 2015; Shen et al., 2018; Stoner et al., 2016; van der Vlist et al., 2012). We here used these four different single EV‐based analysis techniques to gain insight into the physical and physiological interactions between LPPs and EVs. Furthermore, we evaluated the influence of different types of LPPs on the quantitative and qualitative FC analysis of EVs and show the implications of LPP‐EV interactions for EV‐based biomarker profiling.

## 2 MATERIAL AND METHODS

### 2.1 Human lipoprotein particles

Purified human lipoprotein particles, that is, human chylomicrons (0.89 mg/mL, catalogue no. 7285‐1000, Biovision Incorporated), human very‐low‐density and low‐density lipoproteins (catalogue no. 437647‐5MG and 6 mg/mL, LP2‐2MG, Merck Millipore, respectively) were purchased and stored according to the manufacturers’ instructions. For experimental condition we used a fixed volume of 2 μL from each sample.

### 2.2 Human plasma

Blood samples from healthy human donors were collected in sodium citrate at a final concentration of 3.2% (0.105 M). Platelet‐depleted plasma (PDP) was obtained within 120 min after collection by two consecutive centrifugation steps at 2500 × *g* for 15 min at room temperature. After each centrifugation step, the supernatant was transferred to a new sterile plastic tube and the pellet was discarded, after which depletion of platelets was verified with an hemato analyser (0 × 10^4^ plt/μL). Samples were then transferred to 1.5 mL tubes and stored at −80°C until used. Collection of blood was approved by the Ethical Committee of Ghent University Hospital (approval EC/2014/0655). Participants provided written, informed consent.

### 2.3 Preparation of EVs

4T1 murine mammary carcinoma cell line (American Type Culture Collection (ATCC), Manassas, VA) were used as a cellular source to obtain EVs. Cells were maintained in Dulbecco's minimal essential medium (DMEM) supplemented with 10% foetal bovine serum, 100 U/mL penicillin and 100 μg/mL streptomycin (Invitrogen, Carlsbad, CA). Every month cell cultures were tested for Mycoplasma contamination using MycoAlert Plus kit (Lonza, Verviers, Belgium). EVs were prepared from conditioned medium (CM) of the 4T1 cell culture as previously described (Geeurickx et al., 2019; Van Deun et al., 2014; Vergauwen et al., 2017). Briefly, cells were washed once with DMEM, followed by two washing steps with DMEM supplemented with 0.5% EV‐depleted foetal bovine serum. Cells were then incubated at 37°C and 5% CO_2_ with 15 mL DMEM containing 0.5% EV‐depleted foetal bovine serum. After 24 h of culture and when cell confluency was >70%, cell counting was performed with trypan blue staining to assess cell viability (>90%) using an automated cell counter (Countess™, Thermo Fisher Scientific). Conditioned medium was then collected and centrifuged for 10 min at 300 × *g* and 4°C. The supernatant was passed through a 0.45 μm cellulose acetate filter (Corning, New York, USA) and concentrated at 4°C approximately 300 times using a 10 kDa Centricon Plus‐70 centrifugal unit (Merck Millipore, Billerica, Massachusetts, USA). After filtration through a 0.22 μm filter (Whatman, Dassel, Germany), concentrated conditioned medium was used for Optiprep density gradient ultracentrifugation. EVs were characterized following the MISEV criteria (Théry et al., 2018). Optiprep (Axis‐Shield, Oslo, Norway) density gradients (ODG) were prepared as previously described (Geeurickx et al., 2019). In brief, a discontinuous iodixanol gradient was prepared by layering 4 mL of 40%, 4 mL of 20%, 4 mL of 10%, and 3.5 mL of 5% iodixanol in a 16.8 mL open top polyallomer tube (Beckman Coulter, Fullerton, California, USA). One millilitre of concentrated conditioned medium was pipetted on top of the gradient and samples were centrifuged for 18 h at 100,000 × *g* and 4°C using a SW 32.1 Ti rotor (Beckman Coulter, Fullerton, California, USA). Fractions of 1 mL were collected from the top and EV‐rich fractions 9 and 10 (corresponding to a density of 1.10–1.12 g/mL) were pooled for additional purification. Size‐exclusion chromatography (SEC) was performed by using a nylon net with 20 μm pore size (NY2002500, Merck Millipore, Billerica, Massachusetts, USA) was placed on the bottom of a 10 mL syringe (BD Biosciences, San Jose, California, USA), followed by stacking of 10 mL Sepharose CL‐2B (GE Healthcare, Uppsala, Sweden). On top of the SEC column, 2 mL of sample was loaded and eluted with PBS. Fractions of 1 mL were collected and EV‐containing eluates 4–7 were pooled together. Pooled eluates were then concentrated approximately 40 times using a centrifugal filter (Amicon Ultra‐2 10k, UFC201024, Merck Millipore, Billerica, Massachusetts, USA) following the manufacturers’ instructions. Concentrated EV‐eluates were resuspended in PBS to a final volume of 100 μL and aliquoted in eppendorf tubes (20 μL each) and stored at −80°C until further use.

Milk EVs from healthy human donors were obtained from fresh and mature milk collected from three healthy mothers (mean age of 33 ± 5 years and mean lactational stage 3.7 ± 3.8 months). Informed consent was given and this study was approved by the local ethics committee. EVs were isolated and characterized using a fully tailored and validated protocol as described previously, using differential centrifugation, density gradient separation and SEC (Zonneveld et al., 2021). EV‐containing SEC fractions were collected, pooled and aliquoted in eppendorf tubes (20 μL each) for storage at −80°C until further use.

### 2.4 Dot blot analysis

Samples were spotted onto a 0.22 μm pore size nitrocellulose membrane (GE Healthcare) and allowed to dry. Membranes were subsequently blocked with PBS containing 0.5% (w/v) fish gelatin (Sigma‐Aldrich) and 0.1% Tween‐20 and incubated overnight at 4°C in a humidified chamber with primary human anti‐CD9 (Biolegend, catalog no. 312102, dilution 1:1000), human anti‐CD63 (BD, catalog no. 556019, dilution 1:1000) or human anti‐ApoB100 (R&D Systems, catalog no. AF3260, dilution 1:1000). After washing with 0.1% Tween‐20 in PBS, membranes were probed with secondary goat anti‐mouse Polyclonal (Jackson ImmunoResearch catalogue no.115‐035‐044, 1:10000) or donkey anti‐goat Polyclonal antibodies conjugated with HPR (Invitrogen, catalogue no. A16005, dilution 1:5000) and detected using Supersignal West Dura Extended Duration chemiluminescent substrate (Thermo Fisher Scientific). Imaging was performed using a ChemiDoc MP system and data was visualized using Image Lab Software v5.1 (Bio‐Rad, Hercules, CA, USA).

### 2.5 Electron microscopy (TEM)

Purified human lipoprotein particles were deposited on carbonated grids and fixed in 2% PFA in 0.1 M phosphate buffer, pH 7.4. The grids were then embedded in methyl‐cellulose/uranyl acetate 0.4% as described (Corona et al., 2023). All samples were examined with a FEI Tecnai Spirit electron microscope (FEI Company), and digital acquisitions were made with a numeric camera (Quemesa; Soft Imaging System). For image quantification, three images from each sample were counted with sample duplicates. At least *n* = 150 structures were quantified per condition.

### 2.6 Synchronous Rayleigh and Raman scattering

**FIGURE 1:**
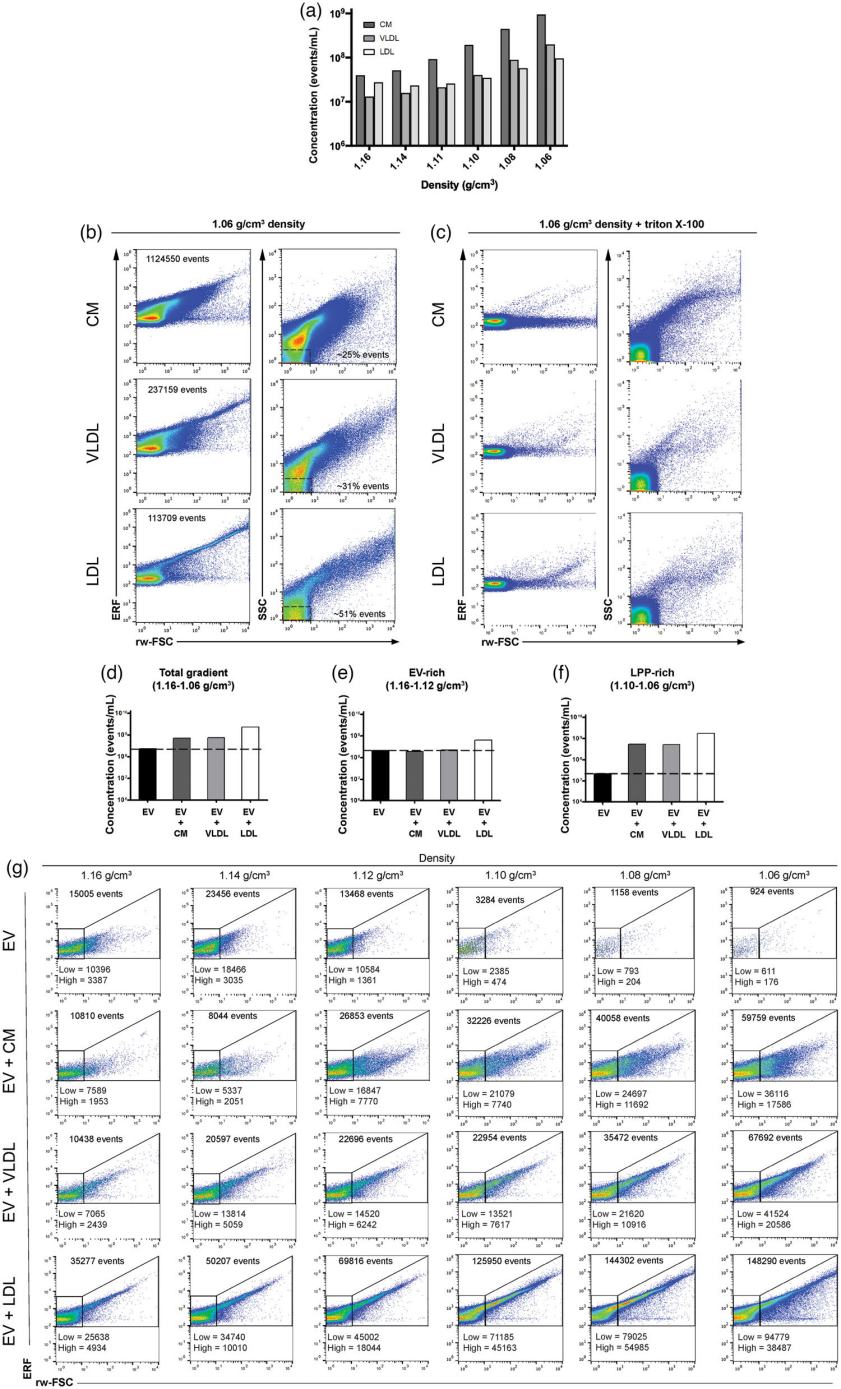
Analysis of generic fluorescence and light scattering profiles of commercial human LPP preparations and mouse EVs in presence or absence of LPPs. (a) Bar graph displaying the concentration of PKH67+ events in LPP, CM, VLDL and LDL preparations respectively, in each density fraction as determined using time‐based flow cytometric analysis. (b) Representative dot plots displaying PKH67 fluorescence in calibrated ERF units versus reduced wide‐angle FSC (rw‐FSC) or SSC versus rw‐FSC of the low density fraction (1.06 g/cm^3^) from CM, VLDL and LDL preparations, respectively. Percentage of the gated low SSC versus rw‐FSC events from the total population is indicated. (c) Representative dot plots displaying PKH67 fluorescence vs. reduced wide‐angle FSC (rw‐FSC) or SSC versus rw‐FSC of the low density fraction (1.06 g/cm^3^) from LPPs after triton X‐100 treatment (final concentration 0.1%). The experiment shown is representative of three independently performed experiments. (d) Bar graph displaying total concentration of PKH67+ events in the density fractions of interest (1.06–1.16 g/cm^3^) (e) Bar graph displaying the total concentration of PKH67+ events in the EV‐rich density fractions (1.12–1.16 g/cm^3^) or (f) in the LPP‐rich density fractions (1.06–1.10 g/cm^3^) from the non‐spiked, CM‐, VLDL‐ or LDL‐spiked EV samples, respectively. (g) Dot plots displaying PKH67 fluorescence in calibrated ERF units versus rw‐FSC of EV‐rich density fractions (1.06–1.16 g/cm^3^) from the non‐spiked, CM‐, VLDL‐ or LDL‐spiked EV samples, respectively. The total number of events for each plot is indicated on top of the plots. The number of the gated rw‐FSC low or rw‐FSC high populations is indicated within the plots. The experiment shown is representative of two independently performed experiments.

For the optical setup measurements, and the characterization of single optically trapped particles, synchronous Rayleigh and Raman scattering acquisition was performed as described (Enciso‐Martinez, Van Der Pol, Hau et al., 2020; Enciso‐Martinez, van der Pol, Lenferink et al., 2020) and briefly in Supporting Materials & Methods. The intensity and wavelength of the Rayleigh‐Raman spectrometer were calibrated as described in Enciso‐Martinez, Van Der Pol, Hau et al. (2020) and briefly in Supporting Materials & Methods. Prior to the Rayleigh and Raman scattering measurements, all samples were diluted in PBS to prevent simultaneous trapping of multiple particles. Preparations of LPPs (CM, VLDL and LDL), 4T1 EVs, Milk EVs and EV‐LPP mixtures were measured. For the LPP‐EV mixtures, a fixed input volume of 5 μL of EVs, corresponding to a nominal amount of 8.7E9 particles for 4T1 EVs and 5E9 particles for milk EVs based on NTA analysis, was spiked with a fixed volume of 2 μL of LPPs (same as used from the samples analysed in Figure 1a–c). The mixed samples were further diluted in PBS to a final volume of at least 300 μL and triplicates were measured alternating between sample types. For each sample type a volume of 50 μL was loaded in the well of a glass slide (BMS Microscopes; 1.0–1.2 mm thick), covered with a glass coverslip (VWR Ltd., thickness No. 1, diameter: 22 mm) and sealed to avoid evaporation. Each glass slide was placed under the microscope objective. The laser focal spot was focused inside the solution ~60 μm below the coverslip. In each cycle of 9.7 s, 256 Rayleigh‐Raman spectra were acquired with an acquisition time of 38 ms per spectrum. The trapped particles were released from the laser focal spot by blocking the laser beam for 1 s. A total of 100 measurement cycles were acquired for each sample (*n* = 21). Hence, a total of 537,600 Rayleigh‐Raman spectra were acquired from which time traces were computed to identify the time intervals corresponding to individual trapping events. A total of 964 individual trapping events were analysed. The computation, segmentation and analysis of the Rayleigh and Raman time traces is described in detail in Enciso‐Martinez, Van Der Pol, Hau et al. (2020) and briefly in Supporting Materials & Methods.

### 2.7 Atomic force microscopy

Samples were prepared in triplicate as described in detail elsewhere (Ridolfi et al., 2022). Briefly, diluted aliquots from samples were left to adsorb at 4°C on poly‐L‐lysine coated glass slides for 30′, gently rinsed with PBS then inserted in a sealed AFM fluid cell. Imaging was performed with scanasyst Fluid+ probes (Bruker, USA) on a Multimode 8 AFM (Bruker, USA) equipped with a Nanoscope V controller and a type‐J piezoelectric scanner in peakforce mode. Maximum applied force was kept under 500 pN and maximum lateral probe velocity under 5 μm/s. Image background subtraction was performed using Gwyddion v2.62 (Nečas & Klapetek, 2012). Image analysis was performed with a combination of Gwyddion and custom Python scripts to calculate the surface contact angles (CA) and equivalent solution diameters (*D*_eq_) of individual particles (Ridolfi et al., 2020), as well as their collective surface density.

### 2.8 Cryo‐electron tomography

The human lipoproteins were prepared in PBS/0.1% aggregate‐depleted BSA, and BSA‐gold 10 nm fiducials (OD_600_ 1). A volume of 2.5 μL was applied on glow‐discharged UltrAuFoil Au200 R2/2 grids (Quantifoil), and excess liquid was removed by blotting for 3 s (blot force 5) using filter paper followed by plunge freezing in liquid ethane using a FEI Vitrobot Mark IV at 100% humidity at 4°C. Electron tomography data were acquired with a 200‐kV Tecnai Arctica transmission electron microscope (Thermo Fisher Scientific) equipped with a Falcon III direct electron detector. Movies were acquired at 53k× magnification using a stage tilt scheme of −60° to 60° in increments of 3° through a total electron dose of 120 e−/Å2 and a defocus target range of −3 to −5 μm. Tilt series were aligned and reconstructed with IMOD using gold‐particles tracking and SIRT, respectively (Mastronarde & Held, 2017).

### 2.9 Fluorescent staining and labelling for high‐sensitivity flow cytometric analysis

Generic staining of particles was performed as previously described (van der Vlist et al., 2012) with some minor modifications indicated below. Briefly, 2 μL of CMs, VLDLs and LDLs or 5 μL of EVs were resuspended in 20 μL PBS/0.1% aggregate‐depleted bovine serum album (BSA) prior to PKH67 staining (Sigma‐Aldrich). The stock solution of aggregate‐depleted BSA (5% w/v) was prepared by overnight centrifugation at 100,000 × *g* (SW28 rotor Beckman Coulter, Fullerton, California, USA; 4°C; κ‐factor 334.2). When antibody labelling was performed, samples were first resuspended in 20 μL PBS/0.1% aggregate‐depleted BSA and incubated with 0.5 μg of Rat anti‐mouse CD9‐PE (Clone: KMC8, IgG2a, κ, Lot. no. 7268877, Becton Dickinson Biosciences) or matched Isotype antibodies (Rat IgG2a, κ, PE‐conjugated, Lot. no. 8096525, Becton Dickinson Biosciences) for 1 h at RT while protected from light exposure. After antibody incubation, samples were stained with PKH67 and cleared from protein aggregates, unbound PKH67 dye and unbound antibodies by overnight bottom‐up sucrose density gradient (SDG) ultracentrifugation at 192,000 × *g* (SW40 rotor Beckman Coulter, Fullerton, California, USA; 4°C; κ‐factor 144.5), according to the previously described protocol (van der Vlist et al., 2012). Gradient fractions of 1 mL were collected and densities were determined by using an Atago Illuminator (Japan) refractometer.

### 2.10 High‐sensitivity flow cytometric analysis

High‐sensitivity flow cytometric analysis was performed with a jet‐in‐air‐based flow cytometer (BD Influx, Beckton Dickinson Biosciences, San Jose (CA)) that is modified and optimized for detection of submicron‐sized particles, and which is fully described in detail previously (van der Vlist et al., 2012). Upon acquisition, all scatter and fluorescence parameters were set to a logarithmic scale. To ensure that each measurement was comparable, a workspace with predefined gates and optimal PMT settings for the detection of 100 and 200 nm yellow‐green (505/515) FluoSphere beads (Invitrogen, F8803 and F8848) was loaded. Upon aligning the fluid stream and lasers the 100 and 200 nm bead populations had to meet the criteria of pre‐defined MFI and scatter values within these gates, where they displayed the smallest coefficient of variation (CV) for side scatter (SSC), reduced wide‐angle forward scatter (rw‐FSC) and FL‐1 fluorescence. The trigger threshold level was set by running a clean PBS sample, thereby allowing an event rate ≤10–20 events/s. For detergent treatment of samples, 1% (v/v) triton X‐100 (SERVA Electrophoresis GmbH, Heidelberg, Germany) was added to a final concentration of 0.1% triton X‐100 and incubated for 30 s at RT prior re‐analysis. When performing quantitative and qualitative analysis of submicron‐sized particles, 50 μL of each fraction was diluted in 950 μL of PBS. Upon loading the sample, the sample was boosted into the flow cytometer until events appeared, after which the system was allowed to stabilize for 30 s. All samples were then recorded for a fixed time of 30 s using BD FACS Sortware 1.01.654 (BD Biosciences). To evaluate the inter‐assay variation, we confirmed reproducibility between two or three independent experiments by measuring the same relative number of fluorescent‐events for the different samples within one experiment; that is, EVs only, LPPs only or spike‐in samples. Particle concentrations were determined by measuring the flow rate of the instrument and correcting the number of detected particles per sample for the dilution and measured time. Fluorescent signals are expressed in calibrated equivalent reference fluorophores (ERF) or molecules of equivalent soluble fluorophores (MESF) units. In between measurements of samples the sample line was washed subsequently with BD FACSRinse (BD Biosciences) and PBS for 5 s. Data analysis was performed using FlowJo Software version 10.0.8. Additional information according to the MIFlowCyt author checklist (Table S1), MIFlowCyt‐EV framework (Table S2) and the calibration of the fluorescence axis (Figure S1) using FlowJo Version 10.5.0 and FCMPASS Version v2.17 is provided in the Supplementary Material & Methods and described (Welsh, Horak et al., 2020; Welsh, Van Der Pol et al., 2020).

### 2.11 Data availability

We have submitted all relevant data of our experiments and all information of 4T1 EVs to the EV‐TRACK knowledgebase (ID: EV190078) (Consortium et al., 2017). Flow cytometry data files are available upon request.

## 3 RESULTS

### 3.1 Fluorescence‐triggered flow cytometry allows the detection of generic membrane‐stained human LPPs in the absence or presence of EVs

To evaluate whether CMs, VLDLs and LDLs are stained with generic membrane dyes used for EV‐staining and detected by fluorescence‐triggered FC, commercially available purified human LPPs were stained with the lipophilic generic membrane dye PKH67, which has been successfully used for fluorescence‐triggered EV detection (van der Vlist et al., 2012). Based on the biochemical properties of PKH67, staining of the single lipid‐layered LPP particles was expected. After incubation with PKH67 bottom‐up sucrose density‐gradient (SDG) ultracentrifugation was performed to evaluate the density at which the three types of LPPs could be detected. Fluorescence‐triggered FC, showed for all LPP‐types that the highest concentration of events was found in the lowest‐density fraction (1.06 g/cm^3^) of the gradient (Figure 1a). The increase in detected events in the low‐density fractions was as expected strongest for the CMs, which are the biggest and least dense LPPs and thus more easily pass the fluorescent threshold as compared to the smaller VLDL/LDL particles. Also at higher densities, corresponding to typical EV‐densities (1.11–1.16 g/cm^3^), PKH67+ events could be detected, which may be caused by the fact that in the experimental set up used not all LPPs fully floated to their density equilibrium yet, or by the presence of co‐isolated EVs. Further analysis of the light scattering showed that the majority of CMs induced the strongest light scattering intensities (Figure 1b, upper panel), whereas LDLs, the smallest particles analysed, induced the weakest light scattering intensities, with more than 50% of the particles displaying low light scattering signals (Figure 1b, bottom panel). Accordingly, VLDL particles, which fall in between CMs and LDLs in terms of size and heterogeneity, showed an intermediate light scattering profile (Figure 1b, middle panel). Although these observations are in agreement with the reported sizes of these LPPs, light scattering signals cannot be interpreted as a direct measurement of nanoparticle‐size without appropriate calibration based on assumed RIs of these particles (Welsh et al., 2017). Interestingly, the LDL sample, and to a lesser extent the VLDL sample, showed a characteristic tail with increasing PKH67 fluorescence (showed in calibrated ERF units, *y*‐axis of the graph), rw‐FSC and SSC signals (Figure 1b, bottom and middle left panels), which can be generated by ‘swarming’ (Groot Kormelink et al., 2016; Libregts et al., 2018) or by the presence of multi‐particle LPP complexes.

Detergent lysis has been suggested as a control to confirm the detection of membrane enclosed EVs in samples and to rule out the detection of contaminants like protein complexes (Osteikoetxea et al., 2015). As LPPs display overlapping biochemical and biophysical features with EVs, we also evaluated the sensitivity of PKH67+ LPPs to detergent lysis. By incubating samples using triton X‐100, a non‐ionic detergent that has been reported to disrupt the lipid membrane of certain EV populations (Osteikoetxea et al., 2015), we observed that upon re‐analysis a great part of fluorescently stained LPPs, and/or their complexes disappeared upon triton X‐100 lysis (Figure 1c). This indicates that these detergent lysis conditions cannot discriminate EVs, LPPs and LPP‐complexes, which is consistent with previous reports also showing detergent lysis of LPPs under varying conditions (e.g. different staining and detection strategies) (Botha et al., 2022; Sódar et al., 2016). After confirming that LPPs can be stained and detected with PKH67, we next investigated whether the presence of different types of LPPs affects the generic staining and detection of EVs. For this we purified EVs from the 4T1 murine mammary carcinoma cell culture supernatant by a combination of ODG ultracentrifugation and SEC (Figure S2a). For FC analysis 4T1 EVs were stained with PKH67, followed by SDG ultracentrifugation. Time‐based quantification of all gradient fractions showed that the peak density fraction of 1.14 g/cm^3^ contained the highest number of events for this 4T1EV preparation (Figure S3a) and serial dilutions confirmed single EV detection (Figure S3b). To investigate how the simultaneous presence of LPPs and EVs in a preparation affects the staining and/or detection of EVs, we selected a fixed input volume of unstained 4T1 EVs (i.e., 5 μL containing a nominal amount of 8.7E9 particles based on NTA analysis) that was spiked with a fixed volume of LPPs (2 μL, similar as used in Figure 1a–c). Next, samples were stained with PKH67 and fractionated by SDG ultracentrifugation for fluorescence‐triggered FC analysis. As expected, the total number of PKH67+ events detected in all density fractions of interest, that is, the sum of PKH67+ events in the range of 1.06–1.16 g/cm^3^ corresponding to both EV‐enriched fractions (1.12–1.16 g/cm^3^) and LPP‐enriched fractions (1.06–1.10 g/cm^3^), was increased in the presence of LPPs (Figure 1d). In the LPP‐rich fractions (1.06–1.10 g/cm^3^), the number of PKH67+ events increased in all spike‐in samples (Figure 1f), whereas purified EVs displayed a very low number of events (Figure 1f), comparable to the procedural controls (Figure S3c). Interestingly, spiking CM or VLDL particles did not affect the total number of PKH67+ events in the EV‐rich fractions (1.12–1.16 g/cm^3^), while spiking with LDL particles led to an increase in the total number of PKH67+ events in these fractions (Figure 1e). This finding suggests the presence of EV‐LDL complexes, which in complexed form are bright enough to pass the fluorescent threshold. Evaluation of the fluorescent and light scattering profiles of the EV‐rich fractions revealed that LPPs also affect qualitative analysis by increasing light scattering and fluorescent signals in the spiked‐in samples (Figure 1g). This was again most apparent in the presence of LDL particles. The PKH67 fluorescence showed in calibrated ERF units and rw‐FSC light scatter signals detected in the LPP‐rich low‐density fractions resembled the previously described PKH67+ LPP pattern (Figure 1b), while in the EV only sample very few events were detected in the low density fraction (Figure 1g, top row). Taken together, our findings demonstrate that the presence of CMs, VLDLs and especially of LDLs in EV samples affects quantitative and qualitative EV‐analysis.

### 3.2 Association between LDL and EVs unveiled by immunoblotting, atomic force microscopy and cryo‐electron tomography

**FIGURE 2:**
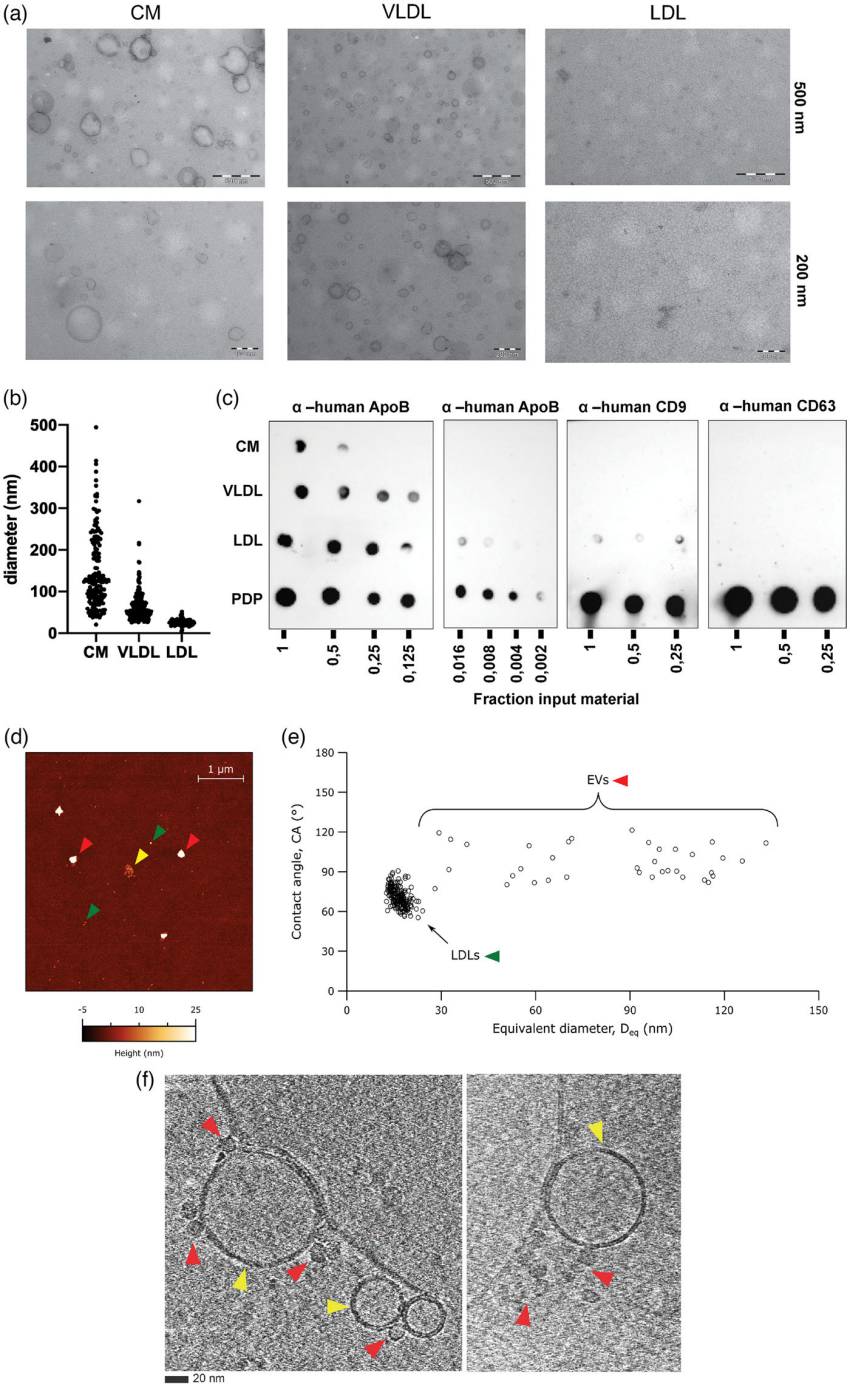
Analysis of purified LPPs by TEM, immunoblotting, atomic force microscopy and cryo‐electron tomography. (a) Analysis of human LPPs by TEM. 4 μL from a 1/10 dilution of commercial preparations of CM (left), VLDL (middle) and LDL (right) particles were loaded onto grids, negatively stained and visualized. Scale bars correspond to 500 nm and 200 nm for each row. (b) TEM quantification of human CM, VLDL and LDL particles showing the size diameter distribution. (c) Dot blot immunodetection of the commercial LPPs next to human platelet depleted plasma (PDP). Serial dilutions were spotted starting from 1 μL LPP stock or PDP from a healthy donor and analysed for the presence of ApoB by using specific anti‐human antibodies against ‐ApoB and the tetraspanins ‐CD9 and ‐CD63. (d) Representative atomic force microscopy (AFM) micrograph of the commercial LDL preparation, showing smaller (green arrow) and larger (red arrow) particles; the smaller particles are occasionally grouped in clusters (yellow arrow). (e) AFM morphometry of individual particles (circles) found in the commercial LDL preparation; each particle is assigned a diameter (*D*_eq_) and a contact angle (CA), which is linked to the particle's mechanical stiffness. All particles in the sample populate zones are compatible with the morphology and nano mechanics of LDLs and EVs. (f) Cryo‐electron tomography of particles present in the commercial LDL preparation. Lipid bilayer enclosed structures (i.e., EVs) are indicated with yellow arrows, while smaller lipid monolayer structures (i.e., LDLs) are indicated with red arrows. Bars = 20 nm for both images.

The profiles from the LPPs revealed by FC, together with previous literature reports showing co‐isolation of LPPs and EVs in EV preparations from blood samples (Karimi et al., 2018; Sódar et al., 2016; Yuana et al., 2014), prompted us to also characterize the commercial LPP preparations in detail. As indicated by the manufacturer these LPP samples obtained from healthy human plasma donors have a >95% purity, which we confirmed with transmission electron microscopy (TEM) showing spheroidal‐shaped LPP particles (Figure 2a). As expected, the CM preparation contained relatively large particles with a rather heterogeneous size range (Figure 2a), LDL‐samples contained the smallest particles with a fairly homogeneous size range distribution of approximately 20 nm (Figure 2a), whereas VLDL particles were found to have a size range in between CM and LDL particles (Figure 2a). Quantification of TEM images confirmed that CMs were largely heterogeneous, but the majority had an average size diameter of 148.2 ± 91 nm. The VLDL preparation contained smaller particles with an average size of 63.5 ± 24 nm and LDLs were 25.3 ± 3.4 nm (Figure 2b). We confirmed the presence of human Apolipoprotein B (ApoB), associated with these LPP types, in the stock solutions of the commercial LPPs and in a human platelet‐depleted plasma (PDP) sample (Figure 2c). Since ApoB‐48 is uniquely expressed in CM, whereas ApoB‐100 is present in VLDL and LDL particles (Feingold & Grunfeld, 2000), the total ApoB signal cannot be used as an absolute measure for comparing these different samples. However, the volumetric‐based analysis of the different preparations shows that the commercial LPP preparations contained less ApoB when compared to the physiological levels of ApoB detected in the PDP sample. Importantly, this indicates that the spike in effects of LPPs on FC analysis of EVs as observed in Figure 1, already occurred at relatively low LPP concentrations, and thus likely to happen as well in physiological blood plasma samples.

To evaluate whether these LPP preparations might contain a small proportion of EVs, we next analysed the commercial LPP preparations by immunoblot using antibodies against two human tetraspanins present in plasma EVs (i.e., CD9 and CD63). As these tetraspanins are genuine transmembrane proteins, LPPs are negative for these proteins. However, a weak signal for human CD9 was detected in the commercial LDL preparation, but not in CM neither in VLDL preparations, suggesting the presence of EVs in the LDL preparation (Figure 2c). To further substantiate this observation, the commercial LDL sample was also analysed by AFM. The resulting micrographs from the LDL preparation evidenced the presence of two distinct classes of particles with different average sizes (Figure 2d). Quantitative morphometry evidenced that the smaller particles had an average diameter (*D*_eq_) of 17 ± 3 nm and an average contact angle (CA) of 71 ± 8°, corresponding to the same set of values previously associated with LDLs (Ridolfi et al., 2022). The larger particles showed a wider dispersion of diameters (∼30–130 nm) and a conserved average CA of 97 ± 12°, in perfect agreement with values previously measured on EVs (Ridolfi et al., 2022). Remarkably, LDL particles sporadically appeared in AFM micrographs as groups of particles in close proximity (Figure 2d), suggesting their association into multimeric aggregates in solution prior to deposition. Technical repeats gave consistent results in terms of the characteristics of individual particles, resulting in the scatterplot showing pools of particles from three separate depositions (Figure 2e). Although the relative abundance of single LDL, clustered LDLs and EVs in successive repeats was variable, suggesting a non‐uniform distribution of particles across the preparation, we quantified the highest observed relative amount of EVs as a 14% of the total number of deposited particles.

By performing cryo‐electron tomography analysis of the commercial LDL sample, we also identified besides the abundant presence of LDL particles (∼20 nm) (Figure 2f, red arrows) bigger sized double lipid layered particles resembling EVs (Figure 2f, yellow arrows). Remarkably, these EVs were not randomly distributed along the grid, but often in close proximity to LDL particles forming complexes of EV‐LDL, suggesting that LDLs might be part of the biomolecular corona of certain EVs. In agreement with our AFM data, we also observed the presence of multimeric‐LDL aggregates, as their layers were physically in contact (Figure 2f, right picture).

### 3.3 Label‐free single particle synchronous Rayleigh and Raman scattering analysis unveiled the physiological formation of EV‐LDL complexes

To investigate whether EV‐LPP complexes can form in solution, we performed optical trapping of particles in suspension and acquired both Rayleigh and Raman scattering signals to detect individual trapping events and to characterize the particles chemical composition, respectively (Enciso‐Martinez, Van Der Pol, Hau et al., 2020; Enciso‐Martinez, van der Pol, Lenferink et al., 2020). We evaluated the scattering profiles of optically trapped particles present in the LPP (i.e., LDL, VLDL and CM) and 4T1 EV preparations, as well as in mixtures of LPP and EV preparations.

**FIGURE 3:**
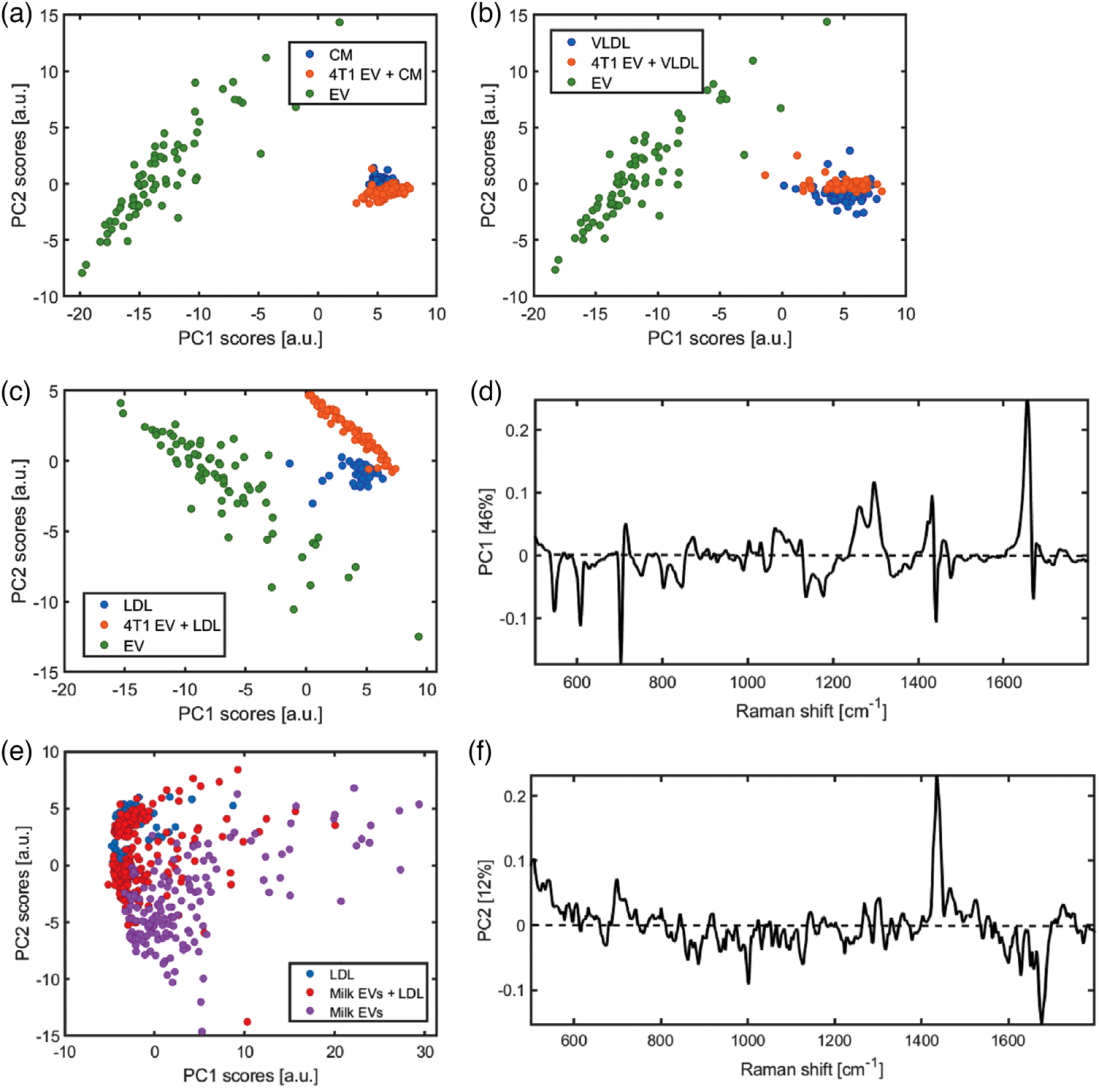
Principal component analysis (PCA) of individual LPP and EV preparations and mixed LPP and EV preparations. First and second principal component scores corresponding to the PCA of (a) CM, 4T1 EVs + CM and 4T1 EVs, (b) VLDL, 4T1 EVs + VLDL and 4T1 EVs, (c) LDL, 4T1 EVs + LDL and 4T1 EVs, (d) First principal component loading corresponding to the PCA of LDL, 4T1 EVs + LDL and 4T1 EVs. The green lines indicate triglyceride bands and the red lines indicate cholesterol bands, (e) LDL, human Milk EVs + LDL and Milk EVs, (f) Second principal component loading corresponding to the PCA of LDL, human Milk EVs + LDL and human Milk EVs. The blue lines indicate lipid bands and the yellow lines indicate protein bands.

Individual trapping events were identified as a step‐wise increase of the Rayleigh signal when plotted over time. By segmenting individual trapping events a Raman spectrum per trapping event was obtained, which was corrected by background subtraction (Enciso‐Martinez, Van Der Pol, Hau et al., 2020; Enciso‐Martinez, van der Pol, Lenferink et al., 2020). To compare Raman spectra of trapped particles, principal component analysis (PCA) was performed on the 4T1 EV preparation, the different LPP preparations, and the EV‐LPP mixtures (Figure 3a–c). Whereas, the particles trapped in the EV‐CM mixture (Figure 3a) or in the EV‐VLDL mixture (Figure 3b) clustered together with respectively CM or VLDL particles only, particles present in preparations of LDL, 4T1 EVs and a mixture of LDL and 4T1 EVs clustered in three separate groups (Figure 3c). Since particles clustering together have similar chemical composition, this indicates that we mainly trapped VLDL or CM particles in the EV‐VLDL mixture and the EV‐CM mixture, respectively. In contrast, particles in the EV‐LDL mixture neither overlaps with particles trapped in the LDL preparation, nor with particles trapped in the EV preparation, suggesting that the particles trapped in the mixed sample have a different chemical composition. As shown in Figure 3c, particles trapped in the EV‐LDL mixture have PC1 scores > 0, similar to LDL particles, and also mainly PC2 scores > 0, resembling the EV sample. This suggests that Raman features from both LDL and EVs contribute to the Raman spectrum of the particles trapped in the mixed sample. Since the difference between LDL and EVs was highest for PC1, we next analysed the PC1 loading to identify the source of these differences. Figure 3d shows PC1 loading displaying positive and negative peaks at wavenumber positions that correspond to triglycerides (green lines) and cholesterol (red lines), respectively. This means that particles with positive PC1 scores, such as particles trapped in the LDL preparation and in the LDL‐EV mixture, have higher triglyceride and less cholesterol contributions to their Raman spectrum than particles with negative PC1 scores, such as particles trapped in the EV only preparation. The high contribution of cholesterol to the Raman spectrum of the EV preparation was further confirmed by Raman bands associated to cholesterol in the high frequency region (2850, 2866, 2888, and 2932 cm^−1^) (Figure S4). Importantly, these results show that EV‐LPP associations are not only induced during isolation and/or staining procedures, but also form spontaneously in solution with label‐free particles. Interestingly, LDL particles are more prone to form complexes with EVs than VLDL and CM.

To investigate whether EV‐LDL associations are EV‐type specific and/or cancer associated only, we performed optical trapping—synchronous Rayleigh and Raman scattering and PCA analysis on EVs purified from human milk in the absence or presence of the commercial LDL preparation. The PCA analysis shows distinct clustering of milk EVs and LDL preparations resulting from their different Raman profiles (Figure 3e). The milk EV‐LDL mixture shows some particles overlapping with either the LDL or milk EV preparations, while other particles in the milk EV‐LDL mixture form a separate cluster. This suggests a mixed Raman profile for such particles, with characteristics that are composed of the Raman profiles of milk EVs and LDL particles. Since the differences between the Raman profiles from the LDL and milk‐EV particles mainly lie along PC2, we next analysed the PC2 loading to identify the source of these spectral differences. Figure 3f shows PC2 loading displaying peaks at wavenumber positions that correspond to cholesterol with lines at 698 cm^−1^ and 1438 cm^−1^ and proteins (yellow lines). In this comparative analysis, the Raman spectra of particles in the LDL preparation show a higher cholesterol contribution than the spectra of particles in the milk EV preparation, which show a higher protein contribution. However, the Raman spectra of the particles from the milk EV‐LDL mixture, which are positioned in between the LDL and milk EV clusters suggests a combination of spectral features, for example, protein and cholesterol contributions, from both LDL and milk EVs. A PCA analysis comparing all sample preparations of Figure 3c–f is shown in Figure S4.Overall, our analyses suggest that EV‐LDL associations can form spontaneously in solution and are not restricted to cancer‐associated EVs or EVs derived from in vitro cell cultures, but also occur with physiological EVs isolated from human milk .

### 3.4 EV‐LPP associations can impact the identification and detection of specific EV markers

Since we found that EV‐LPP associations can form spontaneously in solution, implicating that EV‐LPP complexes are likely present in blood plasma, we further investigated whether such associations can impact the identification and detection of specific EV‐markers by FC. Our experimental set‐up in which mouse‐derived 4T1 EVs were spiked‐in human LPP preparations allowed the use of a murine‐specific CD9 antibody to detect the EV‐marker of interest. We confirmed the exclusive detection of murine CD9+ EVs without cross‐reactivity in human LPP preparations by immunoblotting (Figure S2d), thereby excluding the risk of the detection of human CD9+ EVs already present in the human LPP preparation, as shown in Figure 2. Specific FC detection of purified murine PKH67+CD9+ EVs was confirmed (Figure S3), and the fluorescent intensity was calibrated (Figure S1).

**FIGURE 4:**
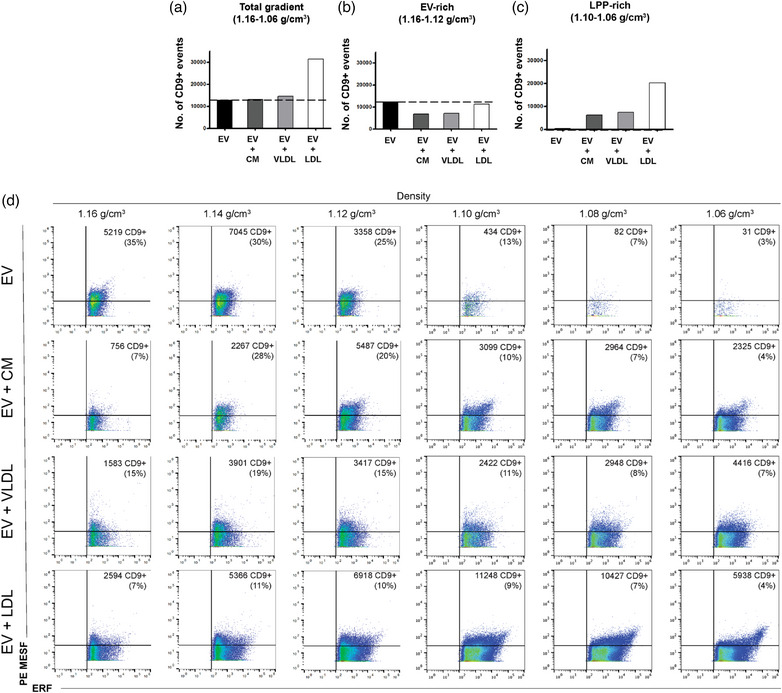
Detection of murine CD9+ EVs in the absence or presence of human CM, VLDL or LDL particles by fluorescence‐triggered high‐sensitivity flow cytometry. (a) Bar graphs displaying the number of PKH67+ CD9+ events in all density fractions of interest (1.16–1.06 g/cm^3^), (b) Bar graphs displaying the number of PKH67+CD9+ events in EV‐rich density fractions (1.16–1.12 g/cm^3^) or (c) in the LPP‐rich density fractions (1.10–1.06 g/cm^3^) from murine EVs in the absence or presence of CM, VLDL or LDL particles. (d) Dot plots displaying CD9+PE events in calibrated PE MESF units (*y*‐axis) versus PKH67+ events in ERF units (*x*‐axis) from murine EVs in the absence or presence of CM, VLDL or LDL particles. The number of gated CD9+ events and its percentage from the total population is indicated for each plot. The experiment shown is representative of two independently performed experiments.

When equal numbers of murine EVs were stained for PKH67 and murine CD9 in the absence or presence of LPPs followed by density gradient centrifugation, different numbers of CD9+ events were detected in time‐based quantitative EV measurements of the total density gradient fractions (1.06–1.16 g/cm^3^) (Figure 4a). In the presence of LDL particles, the total number of CD9+ events were strongly increased, while no or marginal increase of the number of CD9+ events was observed in the presence of CM or VLDL particles, respectively (Figure 4a). In contrast, in the EV‐rich density gradient fractions (1.12–1.16 g/cm^3^) the highest number of CD9+ EVs was detected in the EV sample in the absence of LPPs and the presence of CM and VLDL substantially reduced the number of CD9+ events (Figure 4b). Previously, we showed that the number of PKH67+ events in EV‐rich densities were consistent for EVs in the absence or presence of CM or VLDL (Figure 1e). Hence, the detection of 2‐fold less CD9+ EV in the presence of CM and VLDL (Figure 4b and d) indicates that CM and VLDL significantly affected the labelling and/or detection of CD9+ EVs. Although in the presence of LDL the number of CD9+ events was only marginally decreased (Figure 4b), the % reduction of CD9+ events was strongest (Figure 4d). This reduction in % CD9+ events is caused by the fact that in the presence of LDL the total number of PKH67+ events was increased in EV‐rich fractions (Figure 1e), in contrast to the number of PKH67+ events of EVs in the presence of CM or VLDL that was similar to EVs only (Figure 1e).

Strikingly, a strong increase in the number of CD9+ events was observed in the LPP‐rich densities (1.06–1.10 g/cm^3^) when EVs were analysed in the presence of LLPs, with the strongest effect in the presence of LDL (Figure 4c). Analysis of the corresponding dot plots revealed that CD9+ events showed in calibrated MESF units (y‐axis), especially in the presence of LDL had a distinct profile (i.e., displaying higher PKH67+ fluorescence showed in calibrated ERF units on the *x*‐axis and CD9+ intensities) (Figure 4d), which could be indicative for physical EV‐LDL interactions. Altogether, these data demonstrate that the co‐presence of LPPs in EV‐samples, not only affects generic EV staining but can also impact the staining, enumeration and buoyant density of EV‐subsets labelled for specific EV‐markers.

## 4 DISCUSSION

To explore the use of blood‐based EV‐biomarkers, confounding effects of non‐EV components, such as the abundant and variable presence of LPPs, need to be considered. Circulating EVs are often analysed by FC and recent studies indicated confounding effects of LPPs present in EV preparations in flow cytometric EV analysis (Botha et al., 2022; Simonsen, 2017; Sódar et al., 2016). Based on the partially overlapping properties of EVs and LPPs, optimized sequential biophysical fractionation protocols were developed to separate LPPs from EVs (Karimi et al., 2018; Onódi et al., 2018; Vergauwen et al., 2021). However, in recent studies it has been indicated that also complexes of EV‐LPP can be detected in EV samples (Busatto et al., 2020; Sódar et al., 2016; Yuana et al., 2014).

We here explored, by using 4 orthogonal single‐EV analysis techniques whether EV‐LPP complexes are present in biological samples and whether such EV‐LPP complexes can be formed in solution or are merely a result of the EV isolation and/or labelling methods used. Furthermore, we evaluated how EV‐LPP complexes affect EV analysis by high‐sensitive fluorescence‐based threshold triggered FC. Hereto, we used an optimized PKH67 staining protocol for single EV‐based FC, taking care of artefacts caused by PKH‐aggregates and including procedural controls (van der Vlist et al., 2012). Using a fluorescence‐based threshold triggering FC approach, the detection limit of particles depends on their generic fluorescent staining (PKH67) intensity, allowing only the detection of particles that exceed the set threshold, which in our instrument was calibrated to ∼100 ERF units based on FITC MESF beads (Arkesteijn et al., 2020; Lozano‐Andrés et al., 2021). By using the generic membrane dye PKH67, known to be incorporated by a variety of lipid‐containing components due to its lipophilic nature (Simonsen, 2019), we here show that PKH67 stained commercial LPPs and purified EVs have partially overlapping fluorescent and light scattering signals as measured by FC, albeit the majority of fluorescent events was detected at different buoyant densities, that is, LPP‐rich density fractions (1.06–1.10 g/cm^3^) and EV‐rich density fractions (1.12–1.16 g/cm^3^). Furthermore, in agreement with previous observations our data show that treatment with triton X‐100 could not distinguish between LPPs and EVs, as both showed sensitivity to lysis under the exact same treatment conditions (Sódar et al., 2016).

Due to their size and refractive index, single LDL particles are unlikely to be resolved at the single particle level with the settings used for FC and OT‐RS analysis. However, their aggregates or multimeric LPP complexes can be detected (Sódar et al., 2016). Various reports have investigated and confirmed the formation of such physical LPP aggregates both in vitro and in vivo (Ivanova et al., 2015; Lu & Gursky, 2013), which is in agreement with our own observation of multimeric LPP complexes by cryo‐ET and AFM. Overall, our results clearly indicate the need for specific markers to attribute PKH67+ fluorescent events to LPPs or EVs, which is in accordance to previous observations (Pužar Dominkuš et al., 2018; Simonsen, 2019).

Our spike‐in experiments using purified human LPPs and murine EVs were designed as a proof‐of‐principle study to address the knowledge gap on the impact of LPPs on EV analysis and to specifically investigate the formation of EV‐LPP complexes (Simonsen, 2017). To avoid artefacts resulting from too high concentrations of LPPs in these spike‐in experiments, we intentionally used lower amounts of purified LPPs as compared to platelet depleted plasma samples (determined by immunoblotting for ApoB). Furthermore, the concentration of PKH dye used was not a limiting factor in the staining efficiency of EVs in the EV‐LPP sample during fluorescence‐triggered FC, since we did not find a reduction of the number of PKH67+ events in the EV‐rich fractions while a strong increase in PKH67+ events was observed in the LPP‐rich fractions after EV‐LPP spike‐in. Importantly, the flow cytometric analysis of a specific EV marker (in our experimental set‐up murine CD9) on EVs in the presence of LPPs clearly demonstrated the presence of murine EV‐human LPP complexes in these samples. Notably, by using the murine CD9 antibody, which is not cross‐reactive with human CD9 (Figure S2), human EVs present in the human commercial LPP preparations, as identified by immunoblotting for human CD9, AFM and cryo‐ET, are not contributing to the EV‐specific CD9 signal measured by FC. Consistent with our cryo‐ET observations of the commercial LDL preparation, showing the occurrence of EVs decorated or surrounded by multiple LDL particles, also others have found EV‐associated markers at lower densities as expected in blood plasma (Karimi et al., 2018), and demonstrated the presence of CD9 in purified LPP preparations (i.e., purified HDL) (Simonsen, 2017). decorated or surrounded by multiple LDL particles.

Importantly, our findings are in line with recent reports showing that interactions between EVs and ApoB‐containing LPPs can be observed using various isolation methods and detection techniques (Linares et al., 2015; Takov et al., 2018). However, since these experiments cannot rule out that the formation of LPP‐EV complexes might be induced by the particle isolation, purification and/or staining methods used, we also explored the formation of such complexes in solution. We here demonstrate for the first time with label‐free single particle synchronous Rayleigh and Raman scattering analysis of purified LPPs, purified EVs and their mixtures, that complexes between EVs and especially LDL particles form spontaneous in solution. Furthermore, we show that EV‐LDL complex formation, is not restricted to in vitro generated cancer‐derived EVs but also occurs in the presence of EVs isolated from human milk.

A recent study described the formation of a protein corona around EVs in plasma by using an omics bulk‐based approach (Tóth et al., 2021). Interestingly, the composition of the protein corona described in that study included LPP‐associated apolipoproteins and supports the presence of EV‐LPP complexes which we unveiled by using four different single EV‐based analysis techniques. Only recently potential functional roles have been assigned to the EV corona, including immunomodulation, angiogenesis and skin regeneration (Wolf et al., 2022). Altogether, these observations add a new layer of complexity to EV biology.

Follow‐up studies are needed to address the amount and the nature of EV‐LPP complexes in vivo and to define EV and LPP subsets prone to form such complexes in health and disease conditions. For EV‐based biomarker profiling, our current findings demonstrate the importance of critical sample collection, preparation and experimental design, since the presence or formation of EV‐LPP complexes can impact the detection of EV markers. This feature can hamper, but might also be exploited to identify EV‐subsets of interest.

## AUTHOR CONTRIBUTIONS

E.L.A. designed and performed experiments, analysed data and wrote the original manuscript. A.E.M., A.G, A.R., S.F.W.M.L., C.P., M.J.C.v.H., G.v.N., M.B. and F.V. performed experiments and analysed data. A.H., P.J.P. and C.O. gave conceptual advice. G.J.A.A. supervised and designed the flow cytometric experiments and wrote the original manuscript. M.H.M.W. supervised the research, designed experiments and wrote the original manuscript. All authors critically reviewed and edited the manuscript.

## CONFLICT OF INTEREST STATEMENT

During this study, the Wauben research group, Utrecht University, Faculty of Veterinary Medicine, Department of Biochemistry and Cell Biology, had a collaborative research agreement with BD Biosciences Europe, Erembodegem, Belgium, to optimize analysis of EV using the BD Influx.

## Supporting information

Supporting Information

Supporting Information

Supporting Information

Supporting Information

Supporting Information
