# Supplementary figures and images for: Physical association of low density lipoprotein particles and extracellular vesicles unveiled by single particle analysis
Source: J Extracell Vesicles. 2023 Nov 9;12(11):12376. doi: 10.1002/jev2.12376 (PMC10634195; doi:10.1002/jev2.12376)

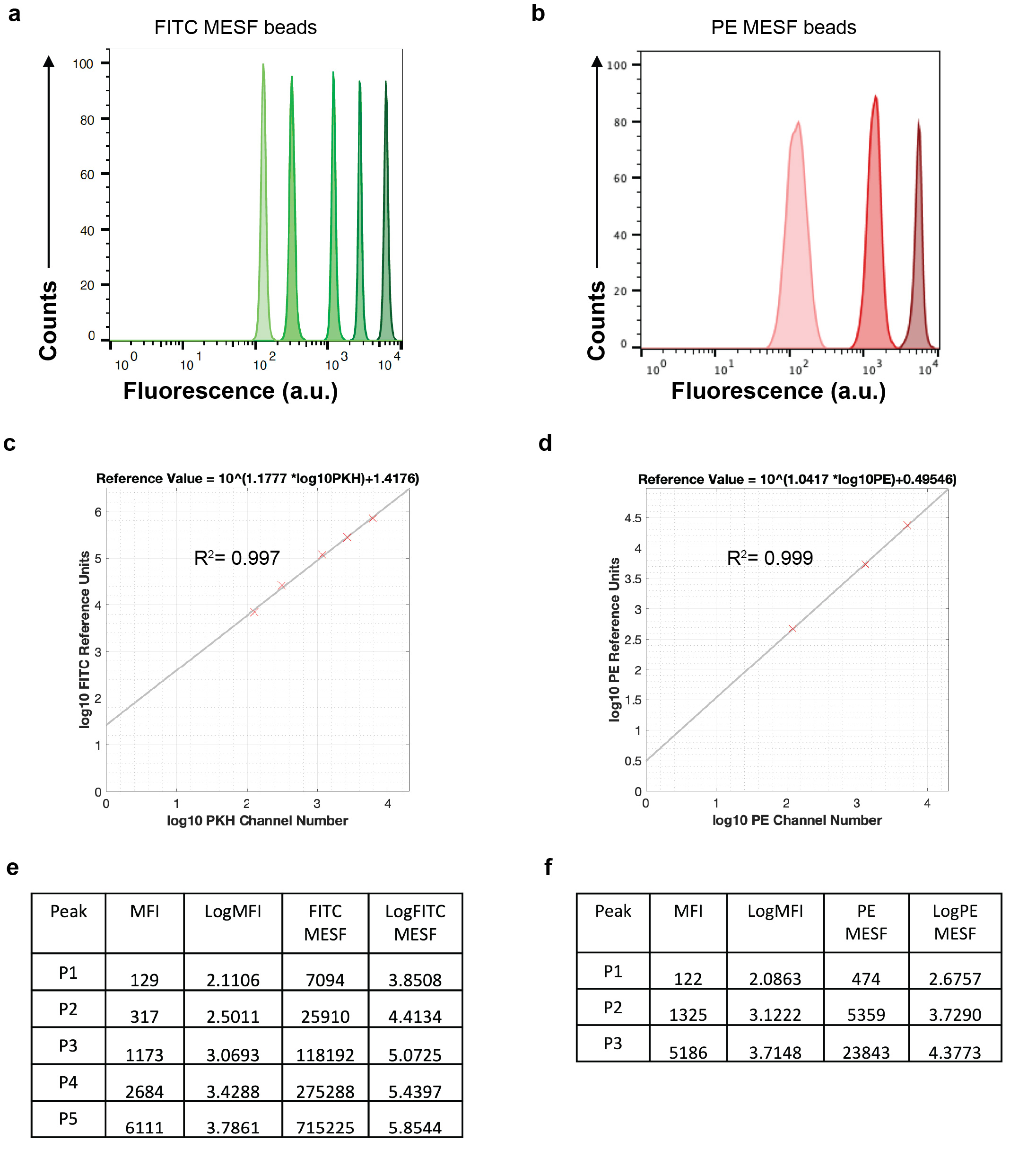

Supplement: Supplementary file 2 — Supporting Information [file JEV2-12-12376-s004.jpg]

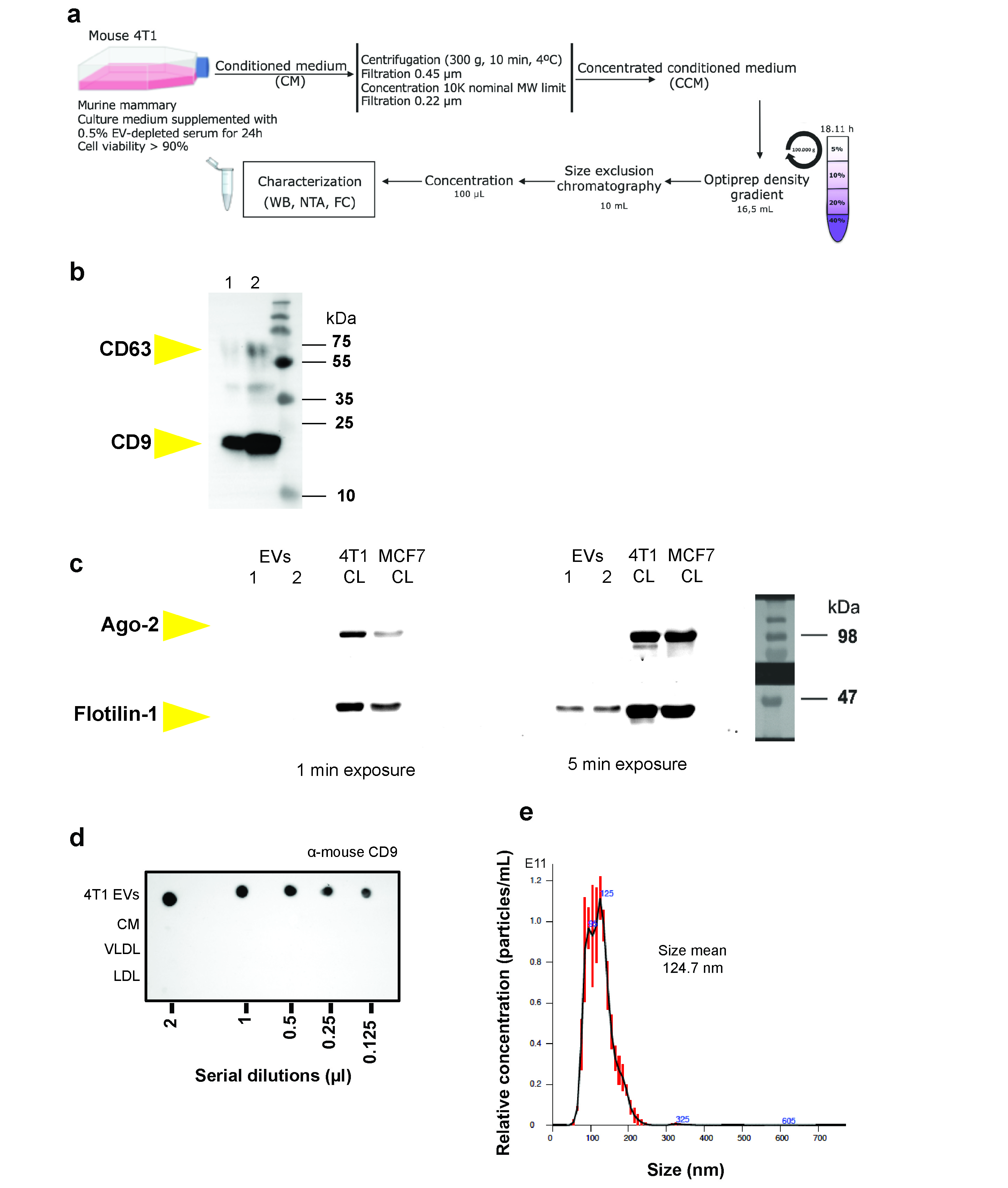

Supplement: Supplementary file 3 — Supporting Information [file JEV2-12-12376-s003.jpg]

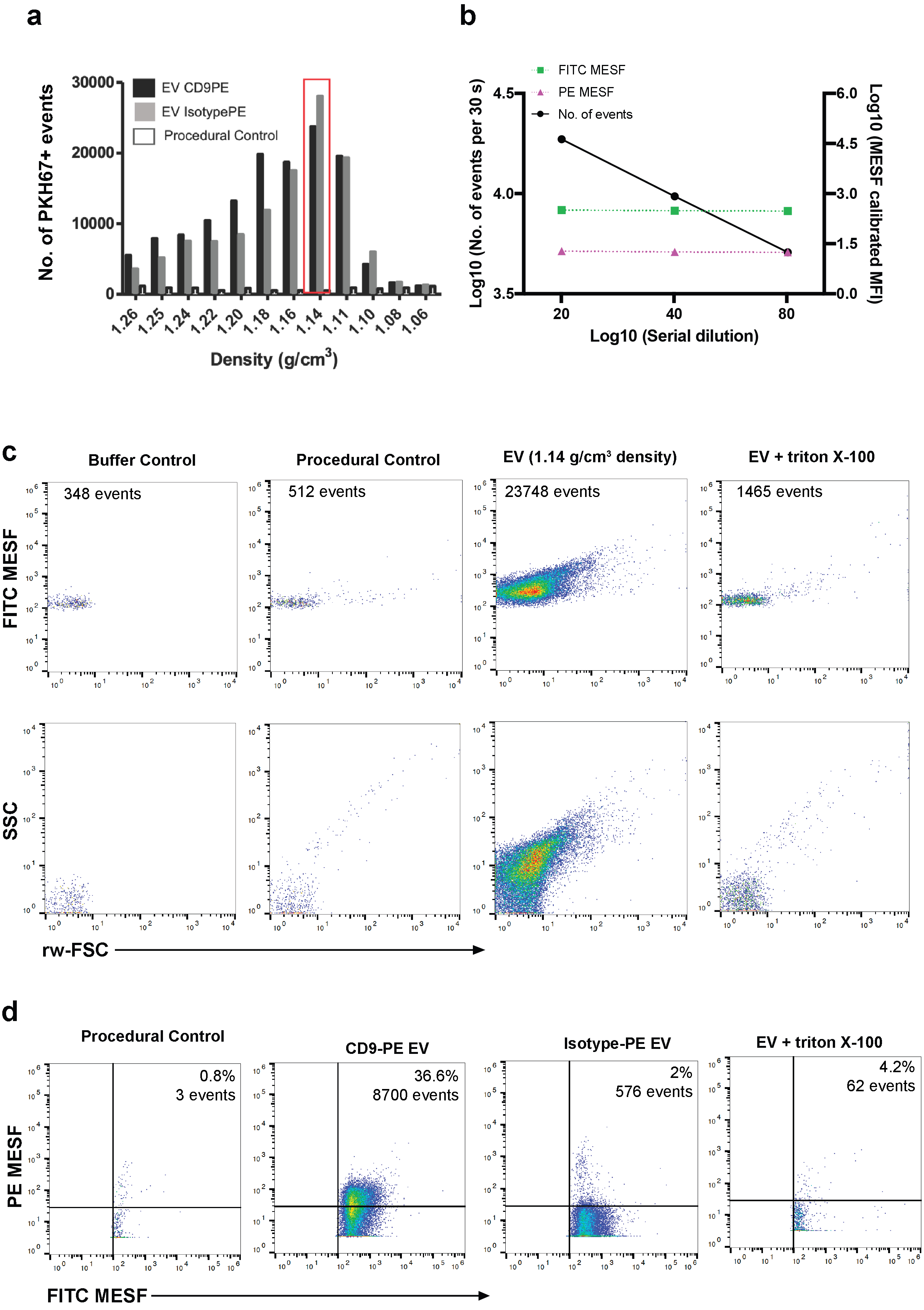

Supplement: Supplementary file 4 — Supporting Information [file JEV2-12-12376-s002.jpg]

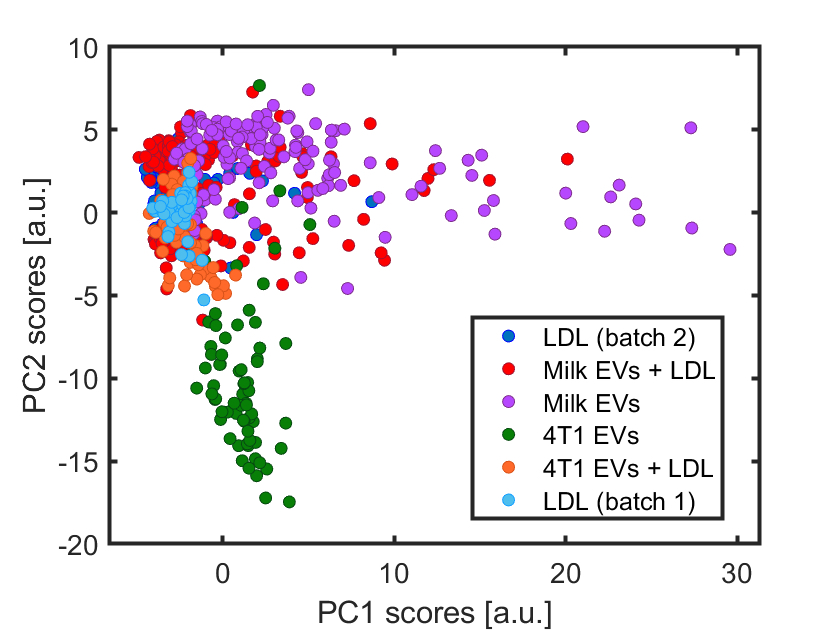

Supplement: Supplementary file 5 — Supporting Information [file JEV2-12-12376-s001.jpg]
